# Risk factors for damage in childhood-onset systemic lupus erythematosus in Asians: a case control study

**DOI:** 10.1186/s12969-018-0271-8

**Published:** 2018-09-10

**Authors:** Jacqueline K. K. Sit, Winnie K. Y. Chan

**Affiliations:** Department of Paediatrics, Queen Elizabeth Hospital, 30 Gascoigne Road, Kowloon, Hong Kong, Special Administrative Region of China

**Keywords:** Systemic lupus erythematosus, Paediatrics, Damage index, Outcome

## Abstract

**Background:**

Accumulated damage is an important prognostic factor in systemic lupus erythematous. However, the pattern of disease damage and its risk factors have not been well studied in childhood-onset systemic lupus erythematosus (cSLE) in Asia. The objectives are to evaluate the pattern of damage and to identify the risk factors for accumulated damage in an Asian group of cSLE.

**Methods:**

A retrospective chart review was conducted on a group of 59 patients with cSLE. Patient demographics and clinical variables were first collected at diagnosis. Over the course of their disease, clinical variables considered as risk factors for damage were also collected. Damage was measured using the Systemic Lupus International Collaborating Clinics/ American College of Rheumatology Damage Index (SDI) for each patient at their last encounter. Based on their SDI scores, patients were then dichotomized to two groups: a group with presence of disease damage (SDI ≥1) and a group with absence of disease damage (SDI score = 0). Clinical variables including age at diagnosis, gender, ethnicity, disease duration, disease manifestations, laboratory values at diagnosis, disease activity at diagnosis and last encounter, major organ involvement, number of lupus flares, major infection, and intensity of immunosuppressive medications were compared between the two groups. Growth failure and estimated glomerular filtration rate (eGFR) were also analysed as secondary outcomes.

**Results:**

After a median disease duration and follow up of 7.8 years, 39 patients (66.1%) had no disease damage while 20 patients (33.9%) had acquired disease damage. Disease damage most frequently occurred in the ocular (15.3%), neuropsychiatric (11.9%) and musculoskeletal (11.9%) domains. The most frequent forms of damage were cataracts (11.9%), and avascular necrosis (unilateral and bilateral combined 10.2%). After controlling for other variables, presence of neuropsychiatric manifestations remained the only statistically significant risk factor for damage. The rate of growth failure in our group of patients was 16%. Patients who experienced growth failure were significantly younger at disease diagnosis. The median age of diagnosis was 10 for those who experienced growth failure, whereas the median age of diagnosis was 13 for those who did not experience growth failure. Despite a high rate of renal involvement in the group (79.7%), renal damage was only seen in 3.2% of the patients. 91.5% of the studied group had normal eGFR of ≥90 ml/min/1.73m^2^ at their last follow up.

**Conclusion:**

This group of patients had a low rate of damage accrual, with one of the lowest rates in renal damage when compared to other cohorts reported. The presence of neuropsychiatric manifestations was identified as the most significant risk factor for disease damage, while the most frequent forms of damage were cataracts and avascular necrosis, which were both related to prolonged steroid use. Despite the limitations of this study, it highlights the need for larger prospective studies to understand the relationship between childhood-onset SLE and its resulting damage.

## Background

Systemic lupus erythematosus (SLE) is a multi-organ autoimmune disease that has wide ranging manifestations. Childhood-onset SLE (cSLE) is notorious for presenting with a more fulminant onset and subsequently runs a more severe and aggressive disease course when compared with adult-onset SLE [1]. Overall survivals of childhood-onset SLE have improved significantly in the past decades. Recently reported 10-year survival rates are around 80 to 90%, a dramatic improvement compared with the 10-year survival rate of 40% that was previously reported 50 to 60 years ago [2–5].

Despite an improved survival, a significant proportion of patients with cSLE still suffer from morbidity due to permanent organ damage [4, 6–12]. Accumulated damage results from the disease process of active inflammation, therapy side-effects as well as comorbid conditions. The importance of evaluating disease damage as a standard assessment in the management of these patients is twofold. Firstly, accumulated damage is one of the important indices used in describing prognosis in SLE [13]. Secondly, it is found that disease damage is significantly associated with lower health-related quality of life [14].

Ethnic differences are known to account for the variability in disease susceptibility and expression in SLE [15, 16]. The objectives of this study are to evaluate the pattern of damage and to identify the risk factors for accumulated damage in our group of childhood-onset SLE. Treatment strategies aimed at reducing permanent organ damage can improve the quality of life of these patients and improve prognosis.

## Methods

### Study population

Patients who were newly diagnosed with SLE between 1 January 1994 and 31 December 2015 and who were subsequently followed up regularly at the department of Paediatrics, Queen Elizabeth Hospital, Hong Kong were included.

All subjects met the diagnostic criteria of SLE either as according to the 1997 Update of the 1982 American College of Rheumatology (ACR) Classification Criteria for SLE [17, 18] or the Systemic Lupus International Collaborating Clinics (SLICC) 2012 classification criteria for SLE [19]. All patients were younger than 18 years at SLE onset with a minimum disease duration of 1 year. Patients who first presented to another hospital at diagnosis or whose charts were incomplete were excluded from the study. Patients who were 18 years old or older at the time of diagnosis, or who had follow up for less than 1 year were also excluded from the study.

Fifty-nine patients were included in the study. Patients records were reviewed from the time of diagnosis to the patient’s last encounter with the paediatrics department, either before transitioning to adult care, loss to follow up or at the end of the study period.

### Study procedures

For each patient, patient demographics and clinical variables were collected at diagnosis. Over the course of disease, clinical variables that were considered as possible risk factors for development of disease damage were collected from medical records. Clinical information regarding various outcomes measures were extracted from the medical records of each patient’s last visit.

### Clinical and laboratory evaluation at diagnosis

Data collected included (i) age at diagnosis, (ii) gender, (iii) ethnicity, (iv) disease manifestations at diagnosis, (v) laboratory data including complete blood count, complement levels (C3, C4), serum albumin, serum creatinine, urinalysis, 24-h urine protein and presence of autoantibodies including ANA, dsDNA, Sm, anti-phospholipid antibodies.

### Risk factors for development of disease damage

#### Disease activity

Disease activity is defined as the reversible manifestations of the underlying inflammatory process. The systemic lupus erythematosus disease activity index (SLEDAI) is an instrument for the evaluation of disease activity in SLE. The SLEDAI has 24 items, and the score of each item is weighted according to the organ system it belongs to. The total score reflects the disease activity of the patient at that juncture [20]. The SLEDAI has been validated for use in childhood-onset SLE [21]. In this study, the SLEDAI was calculated at diagnosis and at the last follow up

#### Medications ever used

Medications used for the treatment of cSLE at any point of the disease course (following diagnosis of disease), including hydroxychloroquine, intravenous methylprednisolone (MP), mycophenolate mofetil or its alternative mycophenolic acid (Myfortic®), cyclophosphamide (CYC), azathioprine, cyclosporine (CsA), intravenous immunoglobulins and rituximab were extracted from the medical records. Cumulative dosage of cyclophosphamide was calculated

#### Major organ involvement

This includes serositis, haematological, renal and neurological disorder. Definitions of each organ involvement are as follows

1. serositis: presence of pleuritis or pericarditis;
2. haematological disorders: presence of haemolytic anaemia with reticulocytosis, leukopenia (< 4000/mm^3^) on two or more occasions, lymphopenia (< 1,500mm^3^) on two or more occasions, thrombocytopenia (< 100,000/mm^3^) in the absence of offending drugs [17];
3. neuropsychiatric manifestations: as characterized by the ACR nomenclature and case definitions for the 19 neuropsychiatric lupus syndromes [22], or a diagnosis of neuropsychiatric SLE by clinician;
4. renal disorder: 24-h urine protein > 0.5 g/24 h, red blood cell casts in urine, or any biopsy proven lupus nephritis. In this study, renal biopsies were classified by the World Health Organization (WHO) classification system for lupus nephritis [19].

#### Lupus flares

Based on clinician’s impression of disease activity, a flare was considered to have occurred when the episode was documented as a flare, or when glucocorticoid or immunosuppressive therapy was changed or escalated [23]. The number of flares that occurred during the study period was collected for each patient. No distinction between renal or non-renal flares were made

#### Major infections

Major infection was defined as any episode of disseminated infection such as septicaemia, affecting major organs such pneumonia, pyelonephritis or meningitis, or those that require hospitalization for treatment. The number of major infections during the study period was collected for each patient

#### Disease duration

Disease duration was defined as the time between the date of diagnosis and the last follow up, calculated in years

### Outcome measures at last follow up

#### Disease damage

Damage is defined as non-reversible change that is not related to active inflammation. The Systemic Lupus International Collaborating Clinics/ American College of Rheumatology Damage Index (SDI) is an instrument that measures accumulated damage in SLE patients, regardless of its cause. It is an index that consists of 41 items, categorized under 12 different organ systems. The SDI has a score range of 0 to 47. Disease damage is ascertained by clinical assessment and present for at least 6 months [13]. It has been validated and proven to be reliable in both adult and paediatric populations. [21] A SDI score for each patient was calculated from data obtained at the last follow up

#### Growth failure

In this study, growth failure was defined as a body height that was below the 3rd percentile for age or body height that had faltered and fallen across at least 2 percentile lines [10, 24]. Growth was assessed based on standards of Hong Kong children [25]

#### Estimated glomerular filtration rate (eGFR)

For patients younger than 18 years old, their eGFR was calculated using the revised bedside Schwartz formula [26]. For patients 18 years old or older, their eGFR was calculated using the Chronic Kidney Disease Epidemiology Collaboration (CKD-EPI) creatinine 2009 equation [27]

## Statistical analysis

The primary outcome was the presence of disease damage. Patients were dichotomized to two groups as according to their SLICC/ACR Damage Index score: one with disease damage and another without disease damage. Presence of disease damage was defined as a SDI score of ≥1, whereas absence of disease damage was defined as a SDI score of 0. Clinical variables were then compared between the two groups to identify potential risk factors for damage.

Normality of data was assessed using the Shapiro-Wilk test. Quantitative values were expressed as medians and interquartile ranges. Qualitative variables were expressed as absolute frequencies and proportions. The Fisher’s exact test was used to compare proportional data, whereas the Mann-Whitney U test was used to compare distributions between non-parametric variables. *P*-values of less than 0.05 were statistically significant. Univariate analysis was performed on the independent variables to look for statistically significant factors. Factors with *P*-values of < 0.05, as well as risk factors considered to be clinically important based on previous research, were included in the multivariate analysis using logistic regression. Factors that were mutually inclusive were not chosen for the multivariate analysis. The Spearman’s correlation was used for measuring associations between categorical variables.

The secondary outcome of interest was growth failure and eGFR. Patients were dichotomized to two groups: ‘no growth failure’ and ‘presence of growth failure’. Clinical variables were compared between the two groups.

All statistical analyses were carried out using IBM SPSS Statistics for Macintosh, Version 24.0.

This study was approved by the Research Ethics Committee of Kowloon Central/ Kowloon East cluster, Hong Kong.

## Results

### Description of included patients

**Fig. 1:**
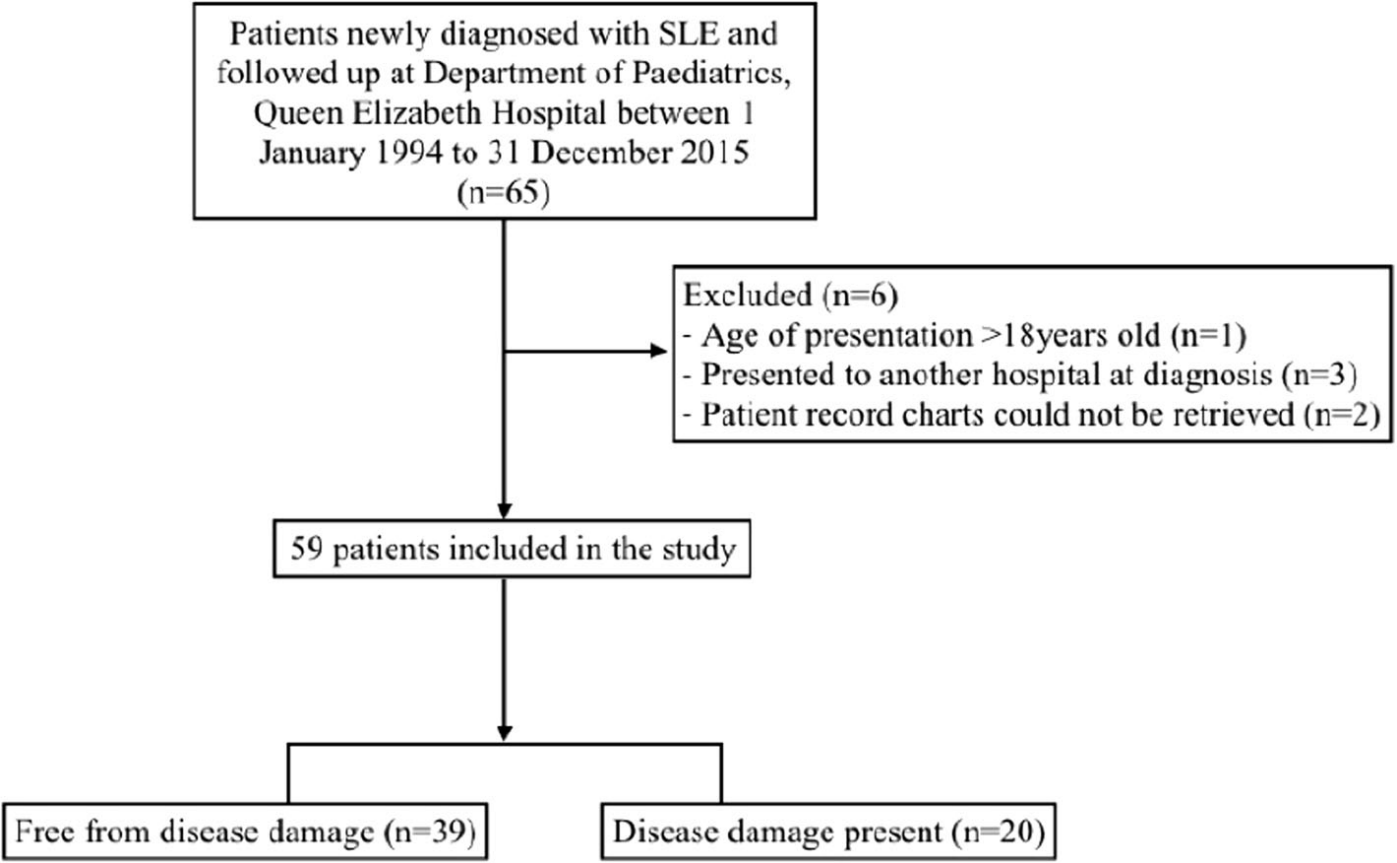
Flowchart of patients included in this study

During the period between 1st January 1995 and 31st December 2015, 65 patients were newly diagnosed with SLE with follow up at Department of Paediatrics, Queen Elizabeth Hospital. Six patients were excluded from the study: one patient was older than 18 years old, three patients presented to another hospital at diagnosis and two patients had incomplete patient records. As a result, 59 patients were included in the final population of the study. There were no deaths during the period studied. This is represented in the flowchart in Fig. 1.

The median age at diagnosis of this present group was 13 years old (interquartile range 12–15), and the female-to-male ratio was 5.6:1. The ethnicity composition was predominantly Chinese (94.9%), and the median duration of follow up was 7.8 years (interquartile range: 5.5–10.1). Renal (79.7%) and haematological (67.8%) were the most frequently involved major organs during the course of disease. The demographic and clinical characteristics of these patients, as well as the different treatment used, are presented in Table 1.

**Table 1:** Demographics and clinical characteristic of the 59 patients included in the study

Patient demographics	
Age at diagnosis (year)	13 [12–15]
Female:male ratio	5.6:1
Ethnicity	
Chinese	56 (94.9%)
Others	3 (5.1%)
Disease duration in years	7.8 [5.5–10.1]
Disease manifestations	
Presence of neuropsychiatric manifestations	9 (15.3%)
Presence of haematological involvement	40 (67.8%)
Presence of renal involvement	47 (79.7%)
WHO class II lupus nephritis	2 (3.3%)
WHO class III lupus nephritis	10 (16.9%)
WHO class IV lupus nephritis	30 (50.8%)
WHO class V lupus nephritis	8 (13.6%)
Presence of serositis	10 (16.9%)
Drug therapies	
Ever use of hydroxychloroquine	36 (61%)
Ever use of intravenous methylprednisolone	28 (31.9%)
Ever use of cyclophosphamide (intravenous or oral)	35 (59.3%)
Ever use of mycophenolate mofetil/mycophenolic acid	21 (35.6%)
Ever use of azathioprine	49 (83.1%)
Ever use of cyclosporine	13 (22%)
Ever use of intravenous immunoglobulin	7 (11.9%)
Ever use of rituximab	1 (1.7%)

Descriptive data represented as median [interquartile range] or frequency (%)

### SLICC/ACR damage index score

At the end of the study period, 39 patients (66.1%) had no disease damage (SDI = 0). Twenty patients (33.9%) had acquired disease damage (SDI ≥ 1). The median SDI score for this group was 0 (range 0–8). The frequency of SDI damage scores as follows: 12 patients had a score of 1, five patients had a score of 2, one patient had a score of 3, one patient had a score of 5, and one patient had a score of 8. Disease damage was most frequently observed in the ocular domain (15.3%), followed by the neuropsychiatric (11.9%) and musculoskeletal (11.9%) domains. The most frequent forms of damage were cataracts (11.9%), and avascular necrosis (unilateral and bilateral combined 10.2%). Details of the frequencies of disease damage in the different items and organ domains are presented in Table 2.

**Table 2:** Frequency of damage in the 12 organ systems and the items of the SLICC/ACR Damage Index

Disease damage by system/ organ at last follow up	Frequency (%)
Ocular	9 (15.3%)
Cataract	7 (11.9%)
Retinal change or optic atrophy	2 (3.4%)
Neuropsychiatric	7 (11.9%)
Cognitive impairment or major psychosis	3 (5.1%)
Seizures requiring therapy for 6 months	3 (5.1%)
Cerebrovascular accident ever	0
Cranial or peripheral neuropathy	1 (1.7%)
Transverse myelitis	0
Renal	2 (3.4%)
Estimated or measured GFR < 50%	0
Proteinuria (nephrotic range proteinuria)	0
End-stage renal disease	2 (3.4%)
Pulmonary ^a^	0
Cardiovascular	2 (3.4%)
Angina or coronary artery bypass	0
Myocardial infarction ever	0
Cardiomyopathy (ventricular dysfunction)	2 (3.4%)
Valvular disease	0
Pericarditis for 6 months	0
Peripheral vascular	1 (1.7%)
Claudication for 6 months	0
Minor tissue loss	0
Significant tissue loss ever	0
Venous thrombosis with swelling, ulceration or venous stasis	1 (1.7%)
Gastrointestinal ^b^	0
Musculoskeletal	7 (11.9%)
Muscle atrophy or weakness	1 (1.7%)
Deforming or erosive arthritis	0
Osteoporosis with fracture or vertebral collapse	0
Avascular necrosis (unilateral)	3 (5.1%)
Bilateral avascular necrosis	3 (5.1%)
Osteomyelitis	0
Skin	3 (5.1%)
Scarring chronic alopecia	0
Extensive scarring or panniculum other than scalp and pulp space	1 (1.7%)
Skin ulceration for > 6 months	2 (3.4%)
Premature gonadal failure	0
Diabetes	0
Malignancy	0

^a^ Includes pulmonary hypertension, pulmonary fibrosis, shrinking lung, pleural fibrosis and pulmonary infarction

^b^ Includes Infarction or resection of bowel below duodenum, spleen, liver or gall bladder ever, mesenteric insufficiency, chronic peritonitis, stricture or upper gastrointestinal tract surgery ever

### Univariate and multivariate analyses of risk factors for disease damage

Clinical variables between the two patient groups, ‘no disease damage’ (SDI = 0) and ‘disease damage present’ (SDI ≥ 1), were compared first using univariate analysis. Disease damage was significantly associated with a younger age at diagnosis (*P* = 0.03), the presence of neuropsychiatric manifestations at any point of the disease course (*P* < 0.01), greater number of major organ involvement (*P* < 0.01), greater number of lupus flares (*P* < 0.01) and greater number of episodes of major infection (*P* = 0.02). The differences in gender, ethnicity, disease duration, laboratory data at diagnosis, presence of other major organ involvement (haematological, renal and serositis), SLEDAI at diagnosis and last visit, use of cyclophosphamide and pulse steroids between the two groups were all not statistically significant.

Number of major organ involvement was excluded from the multivariate analysis as it was inclusive of the presence of neuropsychiatric manifestations. After controlling for other variables in multivariate logistic regression, presence of neuropsychiatric manifestations remained the only significant factor for damage, with an odds ratio of 14.59. These results are presented in Tables 3 and 4.

**Table 3:** Univariate analysis of risk factors associated with disease damage

Risk factors	No disease damage (*n* = 39)	Disease damage present (*n* = 20)	Odds ratio (95% CI)	*p*-value
Patient demographics				
Age at diagnosis	**13 [12–15]**	**12 [10–14.8]**	**0.77 (0.62–0.97)**	**0.03**
Male	5 (12.8%)	4 (20%)	1.7 (0.40–7.20)	0.47
Ethnicity (Chinese)	37 (94.9%)	19 (95%)	1.03 (0.09–12.06)	0.98
Disease duration (years)	7.6 [4.6–11.1]	9.45 [6.4–11.3]	1.08 (0.93–1.26)	0.29
Laboratory data at diagnosis				
Haemoglobin (g/dL)	10.6 [9.5–11.8]	10.6 [6.8–11.8]	0.90 (0.67–1.22)	0.51
White cell count (× 10^9^/L)	5 [3.5–7.0]	3.95 [3.6–5.7]	0.87 (0.70–1.08)	0.20
Platelet (×10^9^/L)	161 [85.5–264.0]	211.5 [96.8–257.5]	1 (1–1.01)	0.40
Serum albumin (g/L)	36 [30.0–40.0]	34 [31.0–36.8]	0.98 (0.92–1.05)	0.64
Serum creatinine (umol/L)	55 [50.0–68.0]	54 [47.0–69.0]	1 (0.97–1.03)	0.78
C3 (g/L)	0.52 [0.3–0.8]	0.41 [0.2–0.5]	0.13 (0.01–1.11)	0.06
C4 (g/L)	0.08 [0.05–0.1]	0.08 [0.04–0.1]	0.03 (0–131.44)	0.40
Anti-dsDNA titre (IU/mL)	210 [83.5–300.0]	300 [213.3–300.0]	1 (0.99–1.00)	0.52
SLEDAI score at diagnosis	12 [7.5–14.3]	11.5 [7.3–18.8]	1.05 (0.97–1.14)	0.22
Disease manifestations over the course of disease				
Neuropsychiatric manifestations	**1 (2.6%)**	**8 (40%)**	**25.33 (2.87–223.62)**	**< 0.01**
Haematological involvement	24 (61.5%)	16 (80%)	2.5 (0.70–8.92)	0.16
Renal involvement	29 (74.4%)	18 (90%)	3.10 (0.61–15.81)	0.17
Serositis	5 (12.8%)	5 (25%)	2.27 (0.57–9.01)	0.25
Number of major organ involvement	**1 [1–2]**	**2 [1.3–3]**	**2.51 (1.34–4.69)**	**< 0.01**
Positivity of Anti-phospholipid antibody	9 (27.3%)	6 (42.9%)	2 (0.54–7.39)	0.30
Treatment related factors				
Cumulative dose of CYC (mg/kg)	70 [0–140]	7.5 [0–115.8])	1 (0.99–1.01)	0.83
Ever use of CYC	20 (51.3%)	15 (75%)	2.85 (0.87–9.38)	0.09
Ever use of intravenous MP	17 (43.6%)	11 (55%)	1.58 (0.54–4.68)	0.41
Events during the course of disease				
Number of clinical flares	**1 [0–4]**	**5 [0.5–8.8]**	**1.25 (1.06–1.46)**	**< 0.01**
Episodes of major infection	**0 [0–1]**	**1 [1–3.8]**	**1.77 (1.11–2.78)**	**0.02**
SLEDAI score at last visit	2 [0–4]	4 [2–7.5]	1.18 (0.98–1.42)	0.08

Descriptive data represented as median [interquartile range] or frequency (%)

Statistically significant variables are highlighted as bold text

*CI* confidence interval, *SLEDAI* SLE disease activity index, *CYC* cyclophosphamide, *MP* methylprednisolone

**Table 4:** Multivariate analysis of risk factors associated with disease damage

Risk factors	Odds ratio (95% CI)	*p*-value
Age at diagnosis	0.88 (0.66–1.18)	0.40
Neuropsychiatric manifestations (during disease course)	**14.59 (1.38–154.16)**	**0.03**
Renal involvement (during disease course)	1.34 (0.20–9.06)	0.76
Total number of flares	1.09 (0.89–1.33)	0.43
Total number of episodes of major infection	1.54 (0.90–2.89)	0.11

The presence of neuropsychiatric manifestations was the most significant risk factor for damage with an odds ratio of 14.59

*CI* confidence interval

The entries in boldface was the only statisitically significant risk factor (*p*-value < 0.05)

### Characteristics of patient with neuropsychiatric manifestations

A total of 9 patients (15.3%) experienced neuropsychiatric manifestations. The median age of diagnosis of SLE in this group of patients was 12 years old (range 7–15), with a female: male ratio of 2:1. Neuropsychiatric manifestations were observed at diagnosis in six patients. Among the nine patients, the neuropsychiatric manifestations included seizures (66.7%), acute confusional state (22.2%), acute psychosis (11.1%), mood disorder (11.1%), cerebrovascular disease (11.1%) and severe headache (11.1%). The types of disease damage experienced by these nine patients included neuropsychiatric (44.4%), ocular (33.3%), musculoskeletal (22.2%), renal (11.1%) and skin damage (11.1%). No significant correlation was found between the presence of neurological disorder and the positivity of anti-phospholipid antibody (*P* = 0.94).

### Growth failure

In the evaluation of this secondary outcome, three patients who were not of Chinese ethnicity were excluded, as the height references of the growth charts used were based on standards of Hong Kong children [25]. Nine out of 56 patients (16%) suffered from growth failure. Comparing possible risk factors of growth failure, age at diagnosis was the only statistically significant independent variable (*P* < 0.01). Patients who experienced growth failure were significantly younger at disease diagnosis (Table 5).

**Table 5:** Univariate analysis of risk factors associated with growth failure amongst the Chinese patients in this study

Risk Factors	No growth failure (*n* = 47)	Growth failure (*n* = 9)	*p*-value ^a^
Patient demographics			
Age at diagnosis	**13 [12–15]**	**10 [7.5–12.5]**	**< 0.01**
Male	8 (17%)	1 (11.1%)	1.00
Disease duration (year)	8.2 [5.5–11.1]	9.3 [5.85–12.5]	0.45
Laboratory data at diagnosis			
Haemoglobin (g/dL)	10.6 [9.5–11.9]	10.55 [9.7–11.5]	0.73
White cell count (×10^9^/L)	4 [3.4–6.7]	6 [3.7–6.9]	0.14
Platelet (×10^9^/L)	183 [100.5–265.0]	198.5 [87.8–261.8]	0.84
Serum albumin (g/L)	35 [30.5–40.0]	37 [32.3–38.8]	0.87
Serum creatinine (umol/L)	55 [50.0–68.5]	52 [43.3–55.5]	0.28
C3 (g/L)	0.48 [0.3–0.7]	0.39 [0.3–0.5]	0.1
C4 (g/L)	0.09 [0.05–0.12]	0.05[0.04–0.08]	0.25
Anti-dsDNA titre (IU/mL)	300 [77–300]	300 [181–300]	0.34
SLEDAI score at diagnosis	12 [8–15]	11 [9–13]	0.72
Disease manifestations over the course of the study period			
Neuropsychiatric manifestations	5 (10.6%)	3 (33.3%)	0.11
Haematological involvement	32 (68.1%)	5 (55.6%)	0.47
Renal involvement	36 (76.6%)	8 (88.9%)	0.67
Serositis	8 (17%)	1 (11.1%)	1.00
Number of major organ involvement	1 [1–2]	2 [1–2.5]	0.64
Treatment related factors			
Cumulative dose of CYC (mg/kg)	27.5 [0–140]	79 [0–121.5]	0.79
Ever use of CYC	26 (55.3%)	7 (77.8%)	0.28
Ever use of intravenous MP	21 (44.7%)	5 (55.6%)	0.72
Events during course of disease			
Number of lupus flares	2 [0–6]	4 [1–6]	0.33
Episodes of major infection	1 [0–2]	1 [0.5–1.5]	0.37

Descriptive data represented as median [interquartile range] or frequency (%)

Statistically significant variables are highlighted as bold text

*SLEDAI* SLE disease activity index, *CYC* cyclophosphamide, *MP* methylprednisolone

^a^ Calculated with Fisher’s exact test or Mann-Whitney U test

### Estimated glomerular filtration rate at last follow up

Two patients had eGFRs of ≤15 ml/min/1.73m^2^. These two patients also had the highest SDI scores in the group, one had a score of 5 and the other had a score of 8. The times from diagnosis to end stage renal disease were 9.8 years and 6.7 years respectively, and renal replacement therapy was initiated for both patients. Three patients had eGFR of 80-89 ml/min/1.73m^2^, while the remainder (91.5%) of the studied group had normal eGFR of ≥90 ml/min/1.73m^2^ during their last follow up.

## Discussion

This study presents the pattern of accumulated damage in a group of 59 Asian patients with childhood-onset SLE. It shows that the presence of neuropsychiatric manifestations is a significant risk factor for damage. Other probable risk factors for disease damage include younger age at diagnosis, greater number of major organ involvement, greater number of flares and major infections.

Damage refers to the irreversible changes that may be related to previous active inflammation, medications or comorbid conditions such as hypertension and atherosclerosis. The SLICC/ACR Damage Index for SLE is an instrument developed for the evaluation of accumulated damage over time. In a clinical setting, it is a simple tool that enables clinicians to monitor patients in a comprehensive and systematic manner, following the course of damage from paediatric age to adulthood. Higher damage index scores early in the course of the disease is associated with a poor prognosis with increased mortality [28]. Furthermore, the SDI is also useful in research settings as it unifies definitions and enables results to be compared between studies.

While the SDI has been shown to be valid in paediatric populations, it has several drawbacks and should be used with caution. Firstly, the SDI does not cover certain damages that are unique to cSLE, such as the impact on growth and development [29]. Hence, a modified paediatric version of SDI has been proposed where growth failure and delayed puberty were incorporated into the score [24]. Some of the items in the SDI signify severe end organ damage and are rarely observed in the paediatric age group, for example, myocardial infarction, pancreatic insufficiency, malignancy or gastrointestinal stricture [24]. This may be explained by the limited duration of follow up in paediatric patients. These patients are transitioned to the care of adult physicians once they enter adulthood, after which these damages may arise. As a result, disease damages that may be clinically relevant yet have not reached the extent as listed in the SDI would not be accounted for and may be underestimated. For the purpose of comparing the results of the present group to previous studies, the original version of the SLICC/ACR Damage Index was used. Growth failure and its risk factors were analysed separately.

**Fig. 2:**
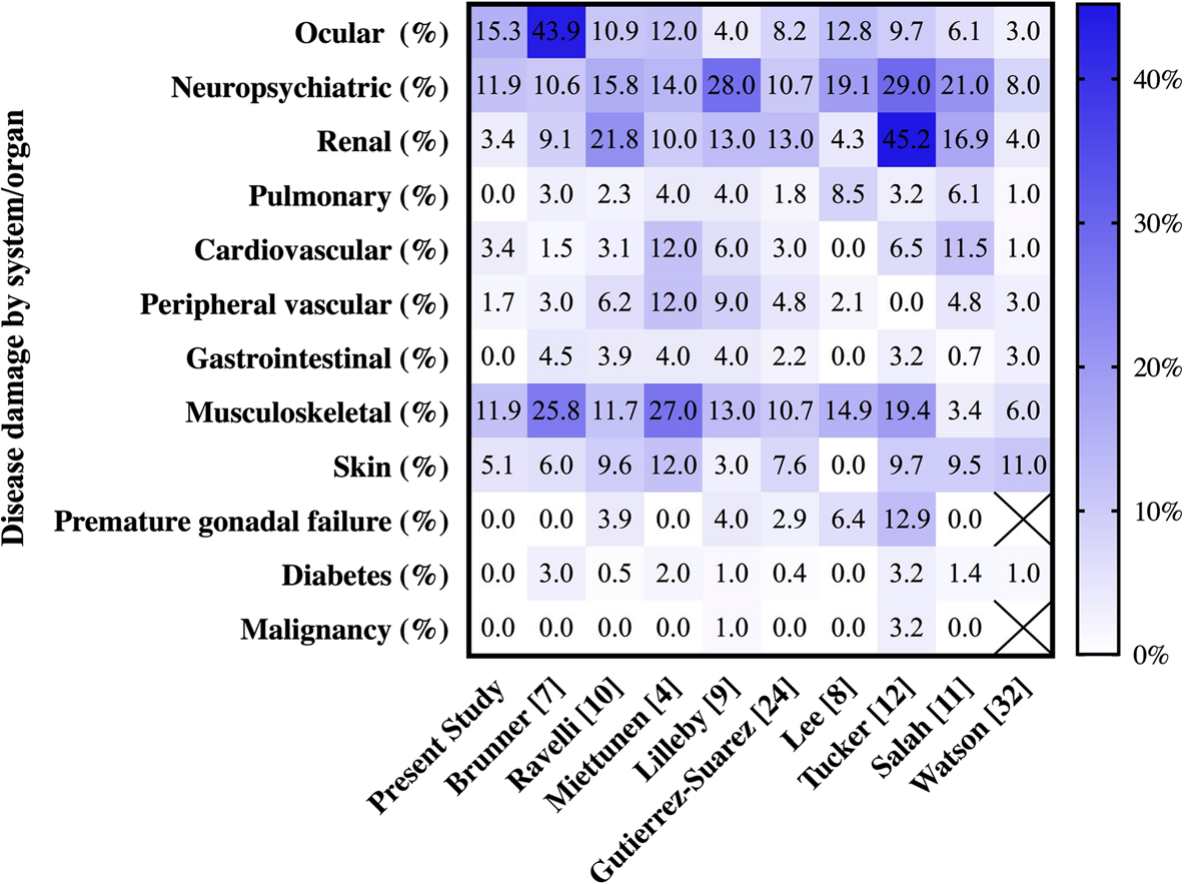
Heat map showing frequency of disease damage in different domains across different studies. In the heat map shown, each column represents a separate study. Each row across represents an organ system/domain. Each figure in the cell represents the percentage of patients with that organ damage in that study. The color of each cell reflects its percentage, with reference to the gradient legend on the right. Among our group of patients, 15.3% had ocular damage, 11.9% had neuropsychiatric damage, 11.9% had musculoskeletal damage, 5.1% had skin damage, 3.4% had renal damage and 3.4% had cardiovascular damage. Percentage of renal damage is among the lowest across the studies

Several authors have looked at the pattern of accumulated damage amongst their cohorts. A summary of the rates and patterns of organ damage in the different series is represented in the heat map shown in Fig. 2. Direct comparison is difficult as the rates were calculated in different cohorts with different study methodology. However, an overall trend can still be appreciated. The most frequently involved organ systems are the ocular, neurological, renal and musculoskeletal systems across most reported studies. Similar to previous studies, this studied group had the highest percentage of damage in the ocular domain (15.3%), followed by the neurological (11.9%) and the musculoskeletal (11.9%) systems.

In contrast to other series, this group had a markedly lower rate of renal damage (3.4%) despite having a high proportion of patients with renal involvement (79.7%). Even when the eGFR at last follow up was calculated, majority of the patients (91.5%) had normal renal functions and there were no patients with nephrotic range proteinuria. Renal failure occurred at 6.7 and 9.8 years from disease onset in the two patients, when both patients had already reached adulthood. Since renal damage is a significant finding amongst adult Asian lupus patients [30, 31], it may be useful to follow up this cohort of patients into adulthood to observe for further significant renal damage that may later arise.

This present group of patients had a median disease duration of 7.8 years. This is longer than most of the previous paediatric series where the median or mean disease duration ranged from 2.7 to 7.2 years [4, 6–8, 10–12, 24, 32]. This is due to the low dropout rates among our patients, and they are followed up into adulthood until referral is accepted by adult physicians. The median SDI score of this group was 0, lower than previous reports where the median or mean SDI scores ranged between 0.93 and 2.3 [4, 6–12]. The percentage of patients with disease damage in this group was 33.9%. It is again lower than most previous reported series where the percentages ranged from 43.9 to 64.5% [4, 6–9, 11, 12, 24]. This may be consistent with some of the previous observations where Asian adult lupus patients have lower rates of damage accrual when compared to other ethnicities [15, 33]. However, a larger sample size would be needed to produce results that can be generalized to the Chinese or Asian populations.

Several studies have been conducted to investigate the risk factors of disease damage in cSLE with different findings. Cumulative disease activity over time and the use of certain drugs, notably high-dose steroids, greater number of cyclophosphamide pulses or ever use of cyclophosphamide, have been implicated as important risk factors of disease damage [7, 9, 10]. Furthermore, frequent severe disease flares and longer disease duration are also associated with presence of damage [6, 7, 9–11].

Neither cyclophosphamide use nor cumulative dosages of cyclophosphamide was found to be a significant risk factor for damage in our patients. This may be explained by the difference in cyclophosphamide regime. As opposed to the National Institute of Health (NIH) protocol (intravenous cyclophosphamide 0.5 – 1 g/m^2^ monthly for 6 months), the more common regimen used in our group is the Euro-Lupus regime (intravenous cyclophosphamide 500 mg every 2 weeks for 3 months) which has a lower cumulative intravenous dose, or a 3-month course of oral cyclophosphamide (at a maximum dose at 2 mg/kg/day) [34]. Lower cumulative cyclophosphamide doses and smaller pulses may have reduced the chances of gonadal failure and infective complications and cyclophosphamide was discontinued once severe complications had occurred. Nonetheless, a larger prospective study and longer observation time would be needed to confirm this.

As in this study, the presence of neuropsychiatric manifestations as a risk factor for damage was observed in two previous studies by Ravelli et al. and Salah et al. [10, 11]. The American College of Rheumatology has developed a standardized nomenclature and case definitions of the 19 neuropsychiatric syndromes of SLE in 1999 to aid clinical research in multi-centre settings [22]. There are wide discrepancies in the reported prevalence of neuropsychiatric manifestations in cSLE, varying between 22 and 95% [35–37]. This reflects the diagnostic difficulties in neuropsychiatric SLE, as subtle syndromes such as headache, mood disorder, or anxiety disorder may be missed in the early stages. The significance of delayed recognition and treatment of neuropsychiatric manifestations in damage accrual is an area that needs further research.

Previous studies have reported rates of growth failure ranging from 10 to 28.3% [9–11, 24], comparable to the rate of 16% in our present group. A large prospective cohort by Rygg et al. has shown that prepubertal and peripubertal children treated with greater than 400 mg/kg cumulative dose of corticosteroids are at risk of having a negative effect on height and pubertal development [38]. Since body height is impacted by genetic, environmental and ethnic factors, the use of parent-adjusted height scores may correct for such confounding effects.

The main limitations of this study were its retrospective nature and the small sample size. Due to its retrospective nature, not all clinical information of interest was available. While some forms of damage were routinely documented (e.g. ophthalmology review and menstruation history), other forms of damages (e.g. muscle weakness, pulmonary symptoms) were not routinely assessed and relied on patients’ self-reporting of symptoms or the physician’s experience and documentation. This could have resulted in underestimation of disease damage amongst the study population.

In the present study, SLE disease activity was assessed at two time points: at diagnosis and at the last follow up. This may not fully reflect the overall disease activity over the course of the follow up. Instead, collection of SLEDAI scores at multiple set time points would give us a better idea of a patient’s overall disease activity over a period of time. The number of disease flares as well as the severity of disease flare would result in more damage. We were not able to retrospectively grade the severity of these flares based on existing definitions as not all the information could be found in chart reviews.

One of the more important variables that we could not assess was the cumulative dosages of steroid over time. While the use of pulse intravenous methylprednisolone (one of the variables studied) reflected the use of high dose steroid in those patients, a more objective measure would be the cumulative dosage of steroid over time. This was especially important as cataracts and avascular necrosis, both related to prolonged steroid use, were the most frequent forms of damage in our group of patients. Some studies have quoted that up to 10–20% of patients with cSLE in Asia suffer from avascular necrosis [3]. Information on the cumulative steroid dosage would help us understand whether the steroid complications in our population was related to higher cumulative dosages of steroids or whether our population of patients were more susceptible to steroid complications. It would also be informative in the evaluation of growth failure for which prolonged steroid is also an important risk factor. Most of the limitations addressed in our study could be overcome by collecting data contemporaneously and prospectively.

## Conclusion

This present study showed that our subjects had a low rate of damage accrual (33.9%) when compared with other studies, despite having a relatively longer median disease duration. Most notably, despite a high rate of renal involvement (79.7%), the rate of renal damage (3.4%) was one of the lowest when compared with other groups. The presence of neuropsychiatric manifestations was identified as the most significant risk factor for disease damage in this group of childhood-onset SLE patients. The most frequent forms of damage were cataracts and avascular necrosis, which were both related to prolonged steroid use.

The findings of this study highlight the need for larger prospective studies on these patients. Prospective systematic collection of data, with particular attention to steroid usage, severity of disease flares, disease activity over time would help to elucidate the relationship between childhood-onset SLE and its resulting damage.

## Abbreviations

ACR: American College of Rheumatology
CKD-EPI: Chronic Kidney Disease Epidemiology Collaboration
cSLE: Childhood-onset systemic lupus erythematosus
CYC: Cyclophosphamide
eGFR: Estimated glomerular filtration rate
MP: Methylprednisolone
NIH: National Institute of Health
SDI: Systemic Lupus International Collaborating Clinics/ American College of Rheumatology Damage Index
SLE: Systemic lupus erythematosus
SLEDAI: Systemic Lupus Erythematosus Disease Activity Index
SLICC: Systemic Lupus International Collaborating Clinics
WHO: World Health Organization
